# Reviving pragmatic theory of theory of mind

**DOI:** 10.3934/Neuroscience.2018.2.116

**Published:** 2018-04-20

**Authors:** Chiyoko Kobayashi Frank

**Affiliations:** 1 School of Psychology, Fielding Graduate University, Santa Barbara, CA, USA; 2 Center for Cognition and Communication, New York, NY, USA

**Keywords:** theory of mind, pragmatics, language, right-hemisphere damage, brain imaging, false-belief, domain-specificity

## Abstract

Theory of Mind (ToM) refers to the ability to attribute mental states to self and others. It has been debated whether or not language capacity precedes ToM in development. Evidence from both neurological and developmental studies suggested that while linguistic capacity is important for ToM understanding, pragmatic component, which is a non-structural part of language, is more important for ToM. Moreover, given that pragmatic component of language is subserved by the right hemisphere of the brain, the evidence also indicates a significant overlap between the neural basis of ToM and that of pragmatic comprehension. The pragmatic theory of ToM, which I aim to revive in this review, firmly links pragmatics to ToM. It regards pragmatic aspects of language and ToM as extensively overlapping functions. I argue that research results from both developmental and neurological studies of ToM are beginning to converge to support this theory. Furthermore, I maintain that the pragmatic theory of ToM provides the best explanation for the seemingly incongruent results from recent child and infant studies on the developmental trajectory of ToM. Lastly, I will discuss whether this theory is in agreement with the domain-specific, the nativist framework, or neither.

## 1. Introduction

Theory of Mind (ToM) refers to the ability to attribute mental states such as beliefs and intentions to self and/or others [1]. To date, ToM or *mentalizing* [2] has been tested with a variety of tasks in several psychiatric conditions. Many of these studies found aberrant patterns of performances in the clinical groups compared to the healthy control groups. Deficits in ToM or *under-mentalizing* has been found in several psychiatric conditions including autism spectrum conditions (ASC) [2]–[4] and schizophrenia [5]–[7], while *over-mentalizaing* has been found in a few conditions such as social anxiety disorder (SAD) [8] and borderline personality disorder (BPD) [9]. A core cognitive feature of individuals with ASC has been described as a difficulty in understanding others' mental states including their false-beliefs [3],[4]. Likewise, a key feature of psychotic symptoms has been described as misrepresentation of one's own and others' intentions [5],[6]. In contrast, it has been found that people with SAD and BPD show more excessive mentalizing than healthy adults [8],[9].

The pragmatic component of language is difficult to define. But to state simply, it involves bringing in general world knowledge, integrating the individual utterances with the context, and making inferences based on one's prior knowledge [10]. Grice [11]–[13] proposed that our everyday conversations are based on a cooperative principle which has four maxims or rules; i.e., quantity, quality, relation or relevance, and manner. For instance, based on the relevance maxim, we generally do not tell someone about a book that we read in the middle of giving the person a direction to a hospital. Depending on the contexts, pragmatics can include understanding humors, ironies, metaphors, and indirect requests [14],[15]. In a broader sense, pragmatics can be thought as *speech acts*, the main purpose of which is to change the attitudes or beliefs of the other conversant [12],[16],[17] and they may be present even in infants [16].

It has long been debated whether or not a person has to have sophisticated language skills in order to have ToM capacity. Some argue that ToM ability precedes language in development [18]–[20], while others maintain that language is a necessary precursor for ToM [21],[22]. A seminal longitudinal study demonstrated that earlier language task competency predicts ToM performance later in development [21]. However, the reverse was not the case; i.e., earlier ToM task competency did not predict later language performance. A meta-analysis of more than a hundred studies that examined the relationship between language and ToM supported the latter, often dubbed “linguistic determinism” hypothesis of ToM [22],[23]. The meta-analysis strengthened the seminal study's results by demonstrating that across different versions of the false-belief task, earlier language capacity independently contributes to later ToM and not the other way around [23]. However, the meta-analysis was limited in that it did not take into account the effects of pragmatics on ToM.

A more recent meta-analysis using a quantitative method found a significant overlap between the neural basis of ToM and that of pragmatic language comprehension [24]. The overlapping brain regions include typical ToM regions; i.e., the medial prefrontal cortex (mPFC) [25]–[29] and the bilateral temporo-parietal junction (TPJ) [27]–[30]. These results are in line with the observation that children with ASC have primary deficits in pragmatic aspects of language [31]–[33]. Children with ASC, for example, might understand a request, “Can you pass me the salt?” as a question about their ability to pass the salt because of the pragmatic deficit. Moreover, it has been shown that people with schizophrenia have impairments not only in ToM but also in understanding figurative, pragmatic aspects of language [6],[34],[35]. These findings point to a close relationship between pragmatics and ToM.

Over the past 10 years, the field of ToM research has changed dramatically. There are now numerous studies of infants aged 6–18 months using various non-verbal ToM tasks claiming that even the pre-verbal infants understand ToM [36]–[39]. These results presented challenge to the commonly accepted hypothesis that ToM develops only around 4 years of age [40]. In order to fill-in the seeming incongruence between results from child studies and those from infant studies of ToM, two main developmental hypotheses were presented. These are the two-system [41] and two-stage [42] hypotheses on the developmental trajectory of ToM (see below for more detailed explanations of these hypotheses). However, the seemingly large gap in the developmental trajectory of ToM can also be explained by the pragmatic hypothesis (as I will explain below).

There are two main aims for this review paper. One is to examine the pragmatic theory of ToM. The pragmatic theory of ToM is not new. Frith, for instance, described schizophrenia as a disorder of pragmatics and thereby, he equated pragmatics to ToM [6],[34]. Likewise, Sperber and Wilson [43] described ToM as a specialized sub-module of pragmatics. Similarly, Malle [44] posited that ToM and pragmatics are co-evolved functions. Most recently, Westra and Carruthers [45] claimed that ToM development is largely dependent upon the pragmatic (i.e., perspective-taking) development. According to my version of the pragmatic theory of ToM, ToM and pragmatic aspects of language are so fused that they cannot be separable. In other words, mine is a stronger position than those mentioned above and is akin to Tom Givón's view [44]. While this theory is preliminary and subject to criticisms and challenges which I will explain in Section 7, results from recent developmental and neuroscientific studies all seem to point to this stronger position. I maintain that research evidence from both developmental and neurological studies including my (and my colleagues') own, has converged to support the long forgotten theory. The other main aim of this review is to delineate the seeming gap in the developmental trajectory of ToM. On this issue, I maintain that the pragmatic theory provides the best explanation for the seeming jump in the development of ToM. Thus my view is in agreement with Westra and Carruthers' [45]. Implications of this theory may or may not challenge the notion of domain-specificity and nativist framework of ToM.

There are several mainstream theories of ToM and pragmatics. The mainstream theories of ToM are modular [46],[47], theory-theory [40],[48], simulation [49],[50], and the aforementioned linguistic determinism theory [22]. Current prominent theories of pragmatics are Grice's speech act (see above) and relevance theory [43]. Previously, these theories have received both validations and invalidations empirically by different researchers [40], [51]–[55]. However, none of these theories has fully delineated the relationship between pragmatics and ToM. In this short review, in order to provide focused arguments, I will discuss some of these theories only in relation to the pragmatic theory of ToM. Readers can refer to other reviews (including my own) [40], [51]–[56] that provide more extensive discussions or comparisons of these theories of ToM and/or pragmatics.

## 2. Evidence from developmental studies

If language is necessary for ToM, it follows that preverbal infants and toddlers do not understand ToM. Results from some studies of ToM in infants demonstrated otherwise. A series of infant studies that used looking-preference paradigm found that even 13–15 month-old infants have the capacity for ToM [36]–[39],[57]. However, these results are seemingly inconsistent with the results from other studies that tested older preschool children using false-belief tasks [40]. In a typical false-belief test, a child is presented with a scenario, in which an object is moved by a protagonist while another protagonist is absent, so that the latter mistakenly believes the object is still in its last location. The child is then asked about the false-belief of the latter protagonist. The purpose of the false-belief test is to assess one's understanding of others' beliefs that may be different from his/her own [3],[4]. The nearly universally observed results are that 4 and 5 year-olds are successful at the false-belief test, while 3 year-olds and older children with ASC are not [3],[4],[44]. Some posit that the seeming gap in the developmental trajectory of ToM can be explained by the two-stage or two-system hypothesis of ToM [41],[42]. According to the former, the verbal, explicit ToM matures only after the non-verbal, implicit ToM [42]. According to the latter, the first subsystem that enables infants to discriminate multiple mental states emerges earlier than the second subsystem that capacitates 4 year-olds to discriminate reality incongruent informational states [41].

In contrary to the above hypotheses, Westra and Carruthers [45] posited that apparent incongruent results can better be accounted for by the development of pragmatics. According to this view, a child's success or failure in the false-belief task depends on whether the child can take the detached third person perspective. Because three year-old children can only take the second person perspective, they respond based on their interpretation of ostensive communicative intention [12],[43]. The ostensive communicative intention (the main purpose of which is to produce a perceptual/cognitive/behavioral change in the recipient) can either be *cooperative* or *competitive* [58]. For instance, children fail the task because they try to help the main character in the story (cooperative bias) or to exhibit their knowledge and show the experimenter where the toy is now (competitive bias). Thus, according to the pragmatic hypothesis, children younger than three fail the false-belief task because of their immature pragmatic interpretation which can either be cooperative or competitive [45],[58].

Ultimately, from the perspective of the full-fledged pragmatic theory of ToM, which I maintain in this review, the above seemingly incongruent results between studies on preschool children and those on preverbal infants are not inconsistent at all. This is because in a broader sense, pragmatic aspects of language include any communicative actions or speech acts such as turn-taking, pointing, cooing, or bubbling that typical infants display [59]. It has been shown that as early as 3 months, infants can coordinate their rhythms with caretakers' rhythms [59]–[61]. The turn-taking, the first prototypical pragmatic ability, appears as early as 3 to 6 months of age [62],[63]. Pointing, another prototype of the speech act [64],[65], emerges around 9 to 12 months of age [66].

Thus, according to the pragmatic theory of ToM, the seemingly incongruent research results from the infants and preschool children are not at all incogruent. This theory views that developmental trajectory of ToM is more continuous and gradual than the two-system or the two-stage hypothesis. At very early points in life, newborns already have the prototypical ToM/speech acts, which will later develop to the full-fledged speech acts. According to this scenario, children learn some prototypical pragmatic ability in early, preverbal stage of life and later these skills develop to an array of speech acts including making requests and conveying refusals, with appropriate inputs from adults [59]. Also, according to this scenario, as children acquire the expressive pragmatic skills along with other aspects of language; i.e., syntactic and semantic aspects, they develop higher levels of speech acts such as understanding of ironies, jokes, metaphors, and false-beliefs.

## 3. Evidence from brain lesion studies

It has been well-established that in human adults, syntactic and semantic aspects of language are specialized in the left hemisphere of the brain [67], while pragmatic aspects of language are specialized in the right hemisphere of the brain [68]–[70]. Several studies on patients with brain injuries showed that right-hemisphere-damaged (RHD) patients have difficulty with understanding non-literal verbal expressions while they have no problem in understanding literal expressions [69],[70]. It has been shown that RHD patients have difficulty with understanding indirect speeches [71], non-literal and figurative expressions including idioms and proverbs [72],[73], humors [74], lies and jokes [75], and sarcasms [76]. Likewise, it has been demonstrated that RHD patients have difficulty in recognizing prosodic cues such as tone of voices and facial expressions [77]–[79]. In a more recent study, the RHD patients with aphasia performed significantly worse in tasks involving formulaic or pragmatic language processing than the left-hemisphere-damaged (LHD) patients with aphasia [80]. On the contrary to the RHD group, the LHD group performed significantly better in tasks that involved literal language processing. Taken together, these results support a dual model of discourse processing which claims that *sentence grammar* and *discourse grammar* are processed in different hemispheres of the brain [81]. The discourse grammar involves non-literal, pragmatic aspects of language and is processed primarily in the right hemisphere of the brain, while the sentence grammar concerns constitutive aspects of language and is processed primarily in the left hemisphere of the brain [81],[82].

Similar results were found in ToM studies on brain damaged patients. These studies consistently demonstrated that while RHD patients fail the false-belief task, LHD patients pass the task [83]–[85]. For instance, a study found a specific impairment in attributing mental states in the second-order format in a group of RHD patients [84]. Similar results were found in another study that used a non-verbal, cartoon-based task in which patients were asked to infer the intentions from geometric shapes. In this study, a group of RHD patients attributed intentions to geometric shapes more inappropriately compared to a group of people without brain damage [86]. Moreover, in a different study, RHD patients performed more poorly than the typical adults, both in a task that required understanding of pragmatic aspects of language and in another task that required ToM capacity [87]. Taken together, these results are in agreement with the pragmatic theory of ToM indicating that both ToM and pragmatics are subserved by the right hemisphere of the brain. However, the brain injury studies are relatively ambiguous about precisely which regions in the right hemisphere are involved in both ToM and pragmatics. In what follows, I will discuss brain imaging studies to address the issue of regional specificity of the neural basis of ToM/pragmatics.

## 4. Evidence from neuroimaging studies

As the results from the developmental studies and brain lesion studies, which I described above, results from brain imaging studies of ToM seem to be in agreement with the pragmatic theory of ToM. Among the ToM brain regions, the medial prefrontal cortex (mPFC) has been the most consistently implicated in ToM [25],[88],[89]. The dorsal mPFC (dmPFC), in particular, has been found to be activated during pragmatic language processing [90]–[92]. For instance, it has been demonstrated that understanding verbal ironies recruits the dmPFC [79],[80]. This region has also been most frequently implicated in both verbal and nonverbal ToM [27],[28],[89],[93],[94]. Moreover, the dmPFC has been shown to be active during both a ToM task and another, different task that requires pragmatic language processing [90]. Results from my study are consistent with this line of results. Through a convergent analysis, I also found converged ToM-specific activity across 56 monolingual and bilingual adult and child participants in the right dmPFC, although the most robust convergence was found in a region more right-lateral than the dmPFC [52].

**Figure 1.**
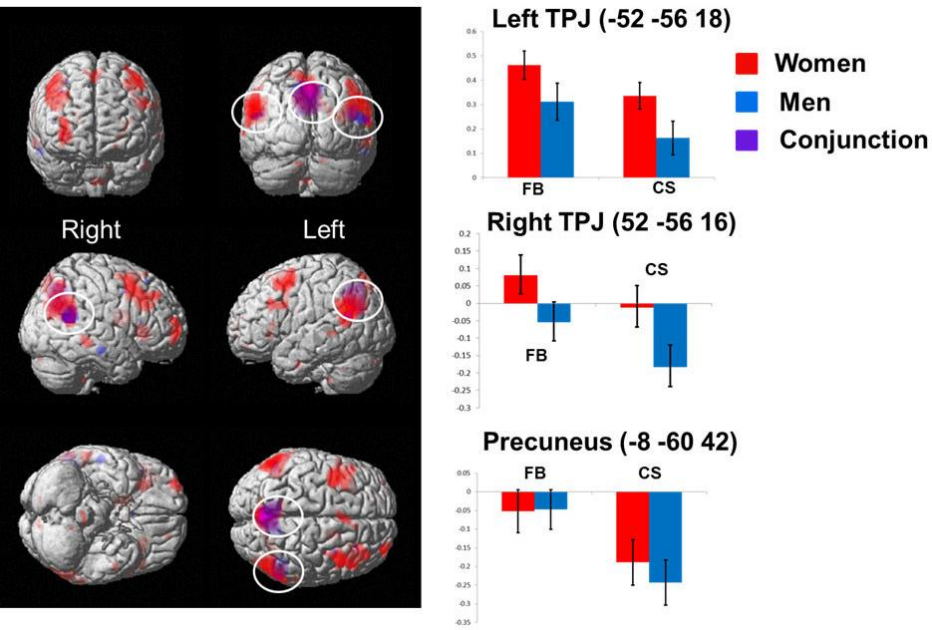
Convergent activity between sexes. The convergent brain activity between sexes was found in the TPJ bilaterally and the precuneus. However, we did not find any sex difference in the false-belief reasoning when we controlled for the coherence or pragmatic aspects in stories by the Coherent Story (CS) condition. Adapted, with permission [29].

As I mentioned earlier, a large meta-analysis across more than a hundred of brain imaging studies found a significant functional and anatomical overlap between ToM and pragmatic language comprehension [24]. These results are consistent with a different, earlier meta-analysis that found an extended overlap between the neural correlates of ToM and those of pragmatics, even after excluding verbal ToM tasks [10]. Most recently, my colleagues and I examined whether pragmatic language or ToM has independent contribution to false-belief reasoning in adults [29]. In this study, we found no evidence for the independent contribution of ToM or pragmatics, in either men or women. Both men and women activated the TPJ during both the coherent story condition (that tapped pragmatic comprehension ability) and false-belief condition equally strongly (Figure 1). As I mentioned earlier, TPJ is another brain region most frequently implicated in ToM [26],[28]–[30],[89]. These results indicate that neural correlates of ToM and those of pragmatic comprehension are very similar if not the same.

Taken together, converged results from developmental, brain lesion and brain imaging studies of ToM and pragmatics are in agreement with the pragmatic theory of ToM. In contrast, as I maintained in my 2010 paper [52], these results are inconsistent with the constitutive language hypothesis or “linguistic determinism” hypothesis of ToM put forward by several others [21],[22]. It is not an aim of this article to argue for that constitutive aspects of language are unimportant for ToM. However, the converged results indicate that these aspects are relatively less important than the pragmatic aspects for ToM.

## 5. Domain-specificity and pragmatic theory of ToM

It might be warranted to discuss whether or not the pragmatic theory of ToM is in agreement with the idea of domain-specificity. Some researchers argued for the modularity or domain-specificity of ToM [20],[55],[95]–[97] and others posited for the domain-generality of ToM [21],[98],[99]. The pragmatic theory of ToM is in line with neither of these extreme positions. Instead, it may be consistent with the *domain-relevance* hypothesis of ToM put forward by Karmiloff-Smith [100]. The domain-relevance hypothesis maintains that a domain-specificity develops from neither a tabula rasa start state nor a modular box state; but from a largely undifferentiated domain-general state to a more differentiated domain-specific state. Research evidence that I will describe below seems to support the domain-relevance hypothesis, but only in the reverse direction; i.e., from the domain-specificity to domain-general.

As I discussed earlier, the pragmatic theory of ToM views pragmatics as an equivalent function to ToM. Even though the pragmatics is a component of language, it is strongly tied to contexts unlike other components such as syntactic and semantic components. Pragmatic capacity or ability to understand communicative intentions cannot develop without appropriate environmental inputs or cultural influences. To support this point, while developmental, longitudinal studies of pragmatics are relatively scarce, there are numerous cross-cultural studies that showed evidence of cultural influences on pragmatics [101]. For instance, there are some significant cultural differences in how politeness [102], self-assertion [103], requests [104], and interrogative communicative acts [103] are recognized and conveyed. Likewise, there are equally numerous studies that demonstrated that mental states are understood and interpreted differently in different cultures [27],[105],[106]. In other words, research evidence has demonstrated that both ToM and pragmatics are significantly malleable and vulnerable to contextual or cultural influences. Thus, they are by no means “informationally encapsulated” or “universal” in Fodor's [96] sense. Therefore, I would argue that neither ToM nor pragmatics is a strictly modular function. However, ToM may become increasingly less modular or domain-specific as a person matures or develops as evidenced in the results of my (and my colleagues') developmental neuroimaging study [89]. In this imaging study my colleagues and I tested bilingual adults and children for their false-belief story understanding. The relevant results are that bilingual adults activated seemingly more dorsal mPFC area during the L1 (Japanese) ToM condition but more ventral mPFC area during the L2 (English) condition. However, bilingual children activated more converged or overlapping mPFC regions of the brain for both conditions. I would argue that it is bilingual adults' ToM that had experienced more contextual or cultural impacts cumulatively over the years. Similar results were found in research on pragmatics. A systematic meta-analysis of brain injury studies on adults indicated that pragmatic capacity is associated with an array of cognitive domains [107]. In this meta-analysis, the authors computed correlations between pragmatics and five key cognitive constructs (i.e., declarative memory, working memory, attention, executive functions, and social cognition). They found significant moderate-to-strong correlations between pragmatics and all of these constructs. These results demonstrate progressively less functional specialization and more generalization in adults' ToM/pragmatics than in children's ToM/pragmatics; therefore, they seem to support the progressively less domain-specificity or the domain-relevance hypothesis of ToM in the reverse direction.

## 6. Nativism and pragmatic hypothesis of ToM

Along with the domain-specificity, Fodor [96] included innateness as one of the criteria for modularity. It may not be an exaggeration to state that many of the aforementioned developmental studies were conducted in order to support the innateness of ToM because one can claim that if a cognitive function appears at a very early point in life, it is more likely that the function is innate. On this issue, my position is similar to the above on the domain-specificity. ToM/pragmatics may be considered as an innate capacity in the sense that some genetic components might be involved in ToM/pragmatics as evidenced in the case of ASC; but ToM/pragmatics may not be innate in the sense that it is considerably malleable or vulnerable to contextual inputs. In fact, as I maintained above, a defining feature of pragmatics is that it has a strong tie to social cognition and hence it is susceptible to contextual and cultural influences. But this does not necessarily rule out the involvement of genetic or biological basis of ToM/pragmatics. As I discussed above, it has been shown that even 13–15 month olds have been shown to have ToM capacity [36]–[39]. Likewise, a prototypical pragmatic capacity (i.e., turn-taking) is even demonstrated by 3 to 6 months-old infants [62],[63].

However, it has been shown that children with ASC will develop ToM with appropriate environmental inputs or trainings that predominantly focus on various speech acts [108]–[110]. For example, a study showed that through an in-home verbal imitation training, toddlers with ASC can increase the usage of single words [109]. Likewise, a recent training/learning study demonstrated that ToM capacity improves in children who showed below average performance in ToM tasks initially [111]. Similar results were found in brain damaged patients. A group of brain damaged patients who initially showed below average performances in ToM tasks improved their performances through an adequate training [112]. Interestingly, however, the results from neither of these studies supported a strong tabula rasa hypothesis. In the former study, only those children who demonstrated some competency in the knowledge access, which is a precursor ability of ToM [113], improved their performances in the ToM task in a microgenetic [114]–[116] way [112]. In the latter study, only those brain damaged patients who were trained with a ToM (not executive functions) training protocol showed improvements [112]. Similarly, it has been demonstrated that augmentative and alternative communication (AAC) intervention, which utilizes a sign-based imitation technique, is not very effective for individuals with ASC who showed severe impairments in vocal skills [117]. Thus, similar to the above debate on the domain-specificity, these results are more in line with the reverse domain-relevance theory which supports neither the strong nativist nor the tabula rasa hypothesis of ToM/pragmatics.

## 7. Challenges and future directions

As other researchers [33],[51],[56] aptly addressed there are several challenges to confirming the present pragmatic theory of ToM. One of the challenges is associated with definitions of both pragmatics and ToM. Currently, neither ToM nor pragmatics is operationally and conceptually defined either clearly or consistently by different researchers. As Cummings [51] aptly expressed, ToM is currently very poorly defined construct. This difficulty seems to stem from the fact that ToM can encompass different functional components including volition, intentions, motivation, and beliefs. To make matters even more complicated, ToM can also include both affective and cognitive components such as empathizing and reasoning [29]. Any of these facets of ToM may overlap with some facets of pragmatics which also embraces both linguistic and intentional/volitional components. For instance, one broad definition of pragmatics is that by Levinson, “relations between language and context that are basic to an account of language understanding” [118]. This definition can encompass mental as well as non-mental aspects. Also, one of ToM's definitions, “ability to perceive intentions of others” [119],[120] is practically identical to a hallmark definition of pragmatics; i.e., “understanding contexts and intentions of speakers” [121]. Moreover, the problem of conflation or semantic mish-mash may extend to developmental studies of ToM. For instance, turn-taking and joint-attention are considered to be precursors for both ToM and pragmatics [59],[123]. These definitional challenges of ToM and pragmatics have generated confusions and lengthy debates among researchers.

To make matters even more challenging, many developmental and neurological studies of ToM have not distinguished between tasks that tap into pragmatics and those that test ToM. For instance, irony and metaphors are often used to test either ToM [123],[124] or pragmatic language comprehension [125],[126]. In these studies, the boundary between ToM and pragmatics is blurred and subject to individual interpretations. Likewise, several researchers pointed out problems associated with the use of false-belief task to test ToM [127],[128]. One of these problems is that current false-belief tasks employ not only ToM but also other related but non-specific cognitive skills such as verbal memory and executive functions [51],[129]. Therefore, it may be necessary for future studies, first, to clearly distinguish ToM from pragmatics by defining each of them operationally and, second, to devise tasks that specifically test the clearly and operationally defined ToM and/or pragmatics.

## 8. Concluding thoughts

In sum, in this article, I argued for the pragmatic theory of ToM. In order to reconcile apparent incongruent results between studies on infants and those on preschool children, two hypotheses were proposed; i.e., the two-system, and the two-stage hypothesis of ToM. As an alternative to these two hypotheses, I proposed the yet third hypothesis; i.e., the pragmatic theory of ToM. I discussed some results from developmental and neurological studies that clearly support the theory. The pragmatic theory of ToM equates ToM with pragmatic capacity which is present from very early, preverbal periods of life in the form of turn-taking. Thus, this theory explains the incongruent results between studies on infants and those on preschool children well by eliminating the requirement of verbal capacity for ToM.

As I delineated above, the pragmatic theory of ToM seems to be consistent with the domain-relevance theory of ToM in the reverse direction. This theory is also in agreement with the microgenetic theory [114],[115] which, I would argue, is a mid-way between the nativist and tabula rasa theories of cognition. ToM/pragmatics may involve some genetic underpinnings; however, this capacity is too malleable and vulnerable to contextual and cultural influences to be considered as a domain-specific or innate capacity. In this review, I also discussed some definitional challenges of both ToM and pragmatics and problems in current task batteries to test either capacity. Until the definitional problem is solved, the pragmatic theory of ToM may remain as preliminary and subject to further refinement. Therefore, more research is definitely needed to confirm the theory. Lastly, once this theory is confirmed, an interesting clinical application drawn from it is that ToM or pragmatic deficits in individuals with brain damages or other psychiatric conditions can be attenuated through adequate trainings that will target either ToM and/or pragmatics.
